# Factors associated with the diagnosis of Brain Death in hospitals in the State of Sergipe

**DOI:** 10.1590/1980-220X-REEUSP-2025-0014en

**Published:** 2025-10-27

**Authors:** José Lucas dos Santos, Gabriella Santos Cisneiros, Jussiely Cunha Oliveira, Eduesley Santana Santos, Fernanda Gomes de Magalhães Soares Pinheiro

**Affiliations:** 1 Universidade Federal de Sergipe, Programa de Pós-Graduação em Enfermagem, Aracaju, SE, Brazil.; 2 Universidade Federal de Sergipe, Lagarto, SE, Brazil.; 3 Universidade Federal de Sergipe, Departamento de Enfermagem, Lagarto, SE, Brazil.; 4 Universidade Federal de Sergipe, Departamento de Enfermagem, Aracaju, SE, Brazil.

**Keywords:** Adult, Brain Death, Epidemiology, Diagnosis

## Abstract

**Objective:**

To analyze the factors associated with the diagnosis of brain death (BD) in hospitals in the State of Sergipe, Brazil.

**Method:**

Qualitative study based on the theoretical-methodological framework of French DiscouCross-sectional study carried out between 2023 and 2024 in four hospitals in Sergipe. Participants were over 18 years of age, of both sexes, with three points on the Glasgow Coma Scale, neurological injury confirmed by brain tomography and absence of at least two brainstem reflexes.

**Results:**

The study included 69 participants and a higher prevalence of individuals from inland cities (65%), with median age of 52 years (42.0–68.0), female (59%), and of mixed ethnicity (62%) was observed. The main neurological causes were stroke and traumatic brain injury. Individuals in older age groups and individuals with cancer are less likely to have BD confirmed. The variable place of residence was significantly associated with the diagnosis.

**Conclusion:**

Integration of well-defined institutional protocols, telehealth consultations, and professional training are recommended as strategies that can lead to faster, safer, and more equitable diagnosis in contexts of structural vulnerability.

## INTRODUCTION

The concept of biological death was reformulated in 1981 based on the Uniform Determination of Death Act (UDDA). This legislation defined death as an event characterized by the irreversible cessation of circulatory and respiratory functions (cardiopulmonary criteria) or by the irreversible cessation of all brain functions, including those of the brainstem^(1)^.

Following the UDDA, Brazil and other countries adopted the concept of Brain Death (BD). Thus, brain death is characterized by the irreversible cessation of the functions of the cerebral hemispheres and brainstem, resulting from the impairment of cerebral circulation, secondary to a known and irreversible injury^(2)^.

The mechanism of BD starts from a decompensation in Cerebral Perfusion Pressure (CPP), which is the result of the difference between Mean Arterial Pressure (MAP) and Intracranial Pressure (ICP). Increased ICP reduces CPP, which consequently compromises circulation throughout the brain. These pressure disorders cause brain damage and favor the onset of progressive edema, due to inadequate oxygenation. This process further aggravates ICP, which can culminate in herniation, interruption of cerebral blood flow and necrosis. In anoxic injuries, hypoxia stimulates the release of cytotoxic substances and the development of cerebral edema^(3)^.

The main causes of BD are related to events that affect the brain, inducing progressive edema and subsequent irreversible damage. Among the most common etiologies, cerebrovascular diseases, Traumatic Brain Injury (TBI), anoxia after Cardiac Arrest (CRA), infections and tumors of the central nervous system stand out^(4)^.

In most countries, the diagnosis of BD is based on neurological assessment of the functions of the entire brain and brainstem, associated with the absence of respiratory movements. In the United States, the diagnosis of BD is guided by guidelines and protocols established by renowned medical organizations^(5)^.

In Brazil, the Federal Council of Medicine (CFM) Resolution No. 2,173/2017 was formulated to define the criteria for the diagnosis of neurological death, making the process more rigorous. Confirmation of this brain injury in the country is based on two clinical examinations with a minimum interval of one hour for patients over two years old, on an apnea test and a complementary examination^(6)^; however, differences permeate its conduct throughout the world. In light of this, global experts have agreed to formulate statements that guide the neurological criteria for BD, aiming to standardize it as much as possible and ensure greater judiciousness in the process^(7)^.

The absence of institutional protocols for the diagnosis of BD, the lack of ICU beds, the unavailability of technological resources, the scarcity of experienced professionals, and incipient professional technical knowledge are strongly associated with difficulties in the diagnosis of BD. Such factors reflect disparities in the understanding and diagnosis of this condition, contributing to gaps in medical education, in the availability of human resources, and in the use of appropriate technologies^(8)^.

In this context, nurses play an essential role in preventing delays and enabling the diagnosis of BD. The continuous work of this professional in the ICU allows for the early identification of neurological worsening, effective communication with the interdisciplinary team and adequate preparation for the exams necessary for diagnosis^(9)^.

Although nurses are recognized in the bureaucratic management of ME care, their effectiveness in providing care and managing the team depends on the development of technical and non-technical skills, improved through experience and continuing education^(10)^. The absence of this improvement compromises their self-confidence and the quality of care, which can lead to delays or failures in the diagnosis of brain death^(11)^.

Thus, the proactive role of nurses, through the team continuing education, improves institutional flows. This professional also plays a strategic role in guiding family members, clarifying the irreversibility of the condition, and reducing resistance that could compromise diagnosis and organ donation. The nurse’s participation in the construction of institutional protocols standardizes behaviors, making the flow more agile and assertive in the confirmation of BD^(11)^.

This study fills an important gap in the literature and provides a basis for future studies in the area, highlighting the structural and educational difficulties that may impact the completion of protocols. For this study, the main question raised was “What clinical, sociodemographic and structural factors are associated with the diagnosis of BD in hospitals in the State of Sergipe”?

Therefore, the study is based on the hypothesis that living in inland cities may be associated with the non-confirmation of the diagnosis of BD, due to multiple factors that hinder the accomplishment of protocols. Among them, the limitations in the technological structure, the unavailability of complementary imaging exams, institutional heterogeneity in terms of care complexity, the shortage of specialized human resources, and deficits in professional training stand out. These factors, together, can contribute to increasing the time needed to confirm the diagnosis.

The objective of this study was to analyze the factors associated with the diagnosis of brain death (BD) in hospitals in the State of Sergipe, Brazil.

## METHOD

### Design of Study

This is a cross-sectional study, guided by the recommendations of the Strengthening the Reporting of Observational Studies in Epidemiology (STROBE).

### Local

This study was conducted in four hospitals in Sergipe. In the capital Aracaju there were two, an emergency hospital (H1) and a philanthropic hospital (H2). In the inland area, a university hospital (H3), located in the city of Lagarto, and a general hospital (H4), located in the city of Itabaiana. The selection of these hospital units was based on CFM Resolution 2,173/2017, which requires neuroimaging to identify the neurological cause in patients eligible for the BD protocol^(10)^. Therefore, all participating hospitals have a 24-hour neuroimaging service and emergency services.

### Population and Selection Criteria

The participants included were those over 18 years of age, of both sexes, who presented a score of three points on the GCS, with neurological injury proven by means of a cranial tomography performed prior to the determination of brain death, and who presented absence of two of the following reflexes: cough, pupillary, or respiratory reflex. Participants whose diagnosis of BD was ruled out, who were on sedation, and those who died before the cranial tomography scan were excluded.

### Sample Size Calculation

The year 2022 was used as a time frame and the 212 protocols initiated in the State and notified to the Organ Procurement Organization (OPO)^(12)^. Assuming a finite population, with a significance level of 5%, a margin of error of 10%, and a prevalence of 50%, the calculated sample size was at least 67 participants, based on the following formula:

$$n=\frac{NZ_{\frac{\alpha }{2}}^{2}p(1-p)}{e^{2}(N-1)+Z_{\frac{\alpha }{2}}^{2}p(1-p)}$$

Where *n* is the sample size, *N* is the population size, *p* is the expected proportion, *e* is the margin of error and $Z_{\frac{\alpha }{2}}^{2}$ is the square of the normal distribution score associated with the significance level *α*.

$$n=\frac{212\times {\left(1,965\right)}^{2}\times 0,5\times (1-0,5)}{{\left(0,10\right)}^{2}(212-1)+{\left(1,965\right)}^{2}\times 0,5\times (1-0,5))}\approx 67$$

### Data Collection

Data collection took place from August 2023 to August 2024 and was performed through interviews with the participants’ family members and legal guardians, analysis of records, and patients’ medical records. The collection instrument was structured and developed by the main author, based on pre-existing forms from the OPO, and had open and closed questions.

The team responsible for data collection consisted of undergraduate nursing students, postgraduate students, and volunteer professionals. All members received theoretical training on the topic, on the objectives of the study, the eligibility criteria for participants, and on how to complete the data collection instrument. To calibrate the researchers, test collections were carried out in the four hospitals, aiming to evaluate the standardization of the collections and the effectiveness of the instrument in obtaining the data necessary for the study.

**Figure 1:**
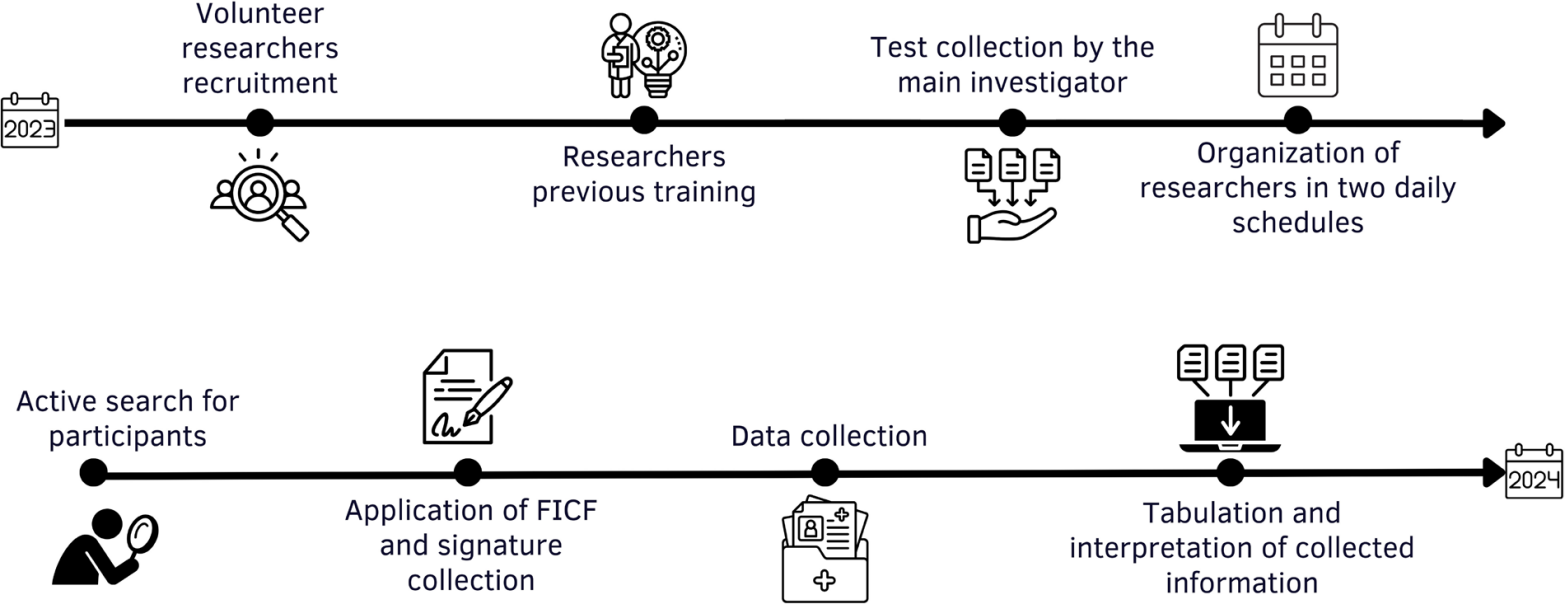
Data collection system.

The interviews allowed us to obtain information on sociodemographic variables, such as race, marital status, place of birth, level of education, number of children, occupation, and income, in addition to the history of previous illnesses, when these data were not recorded in the participants’ medical records. The data collection steps are detailed in Figure 1.

Participants were selected through an active search in the emergency units and Intensive Care Units of the institutions. Following the verification of the inclusion criteria, the Informed Consent Form was applied to the family member or legal guardian through an interview.

### Data Analysis and Treatment

Statistical analysis was performed using measures of central tendency (mean and median), dispersion (standard deviation and interquartile range), and distribution (absolute and percentage frequency).

The association between categorical variables was assessed using the Chi-square test, with Fisher’s exact test being applied when appropriate. To compare the medians between two or more independent samples, the Wilcoxon-Mann-Whitney, Kruskal-Wallis tests were used and to specifically identify which groups differed from each other, the Dunn’s test was used*,* applied as *post hoc*.

Logistic regression was used to model the relationship between predictor variables and binary outcomes, using crude (with only the variable of interest), adjusted, and multivariate models, considering only significant variables in the final model. The selection criteria adopted were significance lower than 0.2, the absence of separation phenomena, and the absence of multicollinearity. The variable selection method used was backward.

Statistical analyses were conducted in the R programming environment (version 4.4.0) (R CORE TEAM, 2023), adopting a significance level of 5%.

### Ethical Aspects

This study was approved by the Research Ethics Committee (*CEP*) of the Universidade Federal de Sergipe on June 21, 2023 under CAAE number: 68731823.3.0000.5546 and opinion number 6,132,287.

## RESULTS

**Figure 2:**
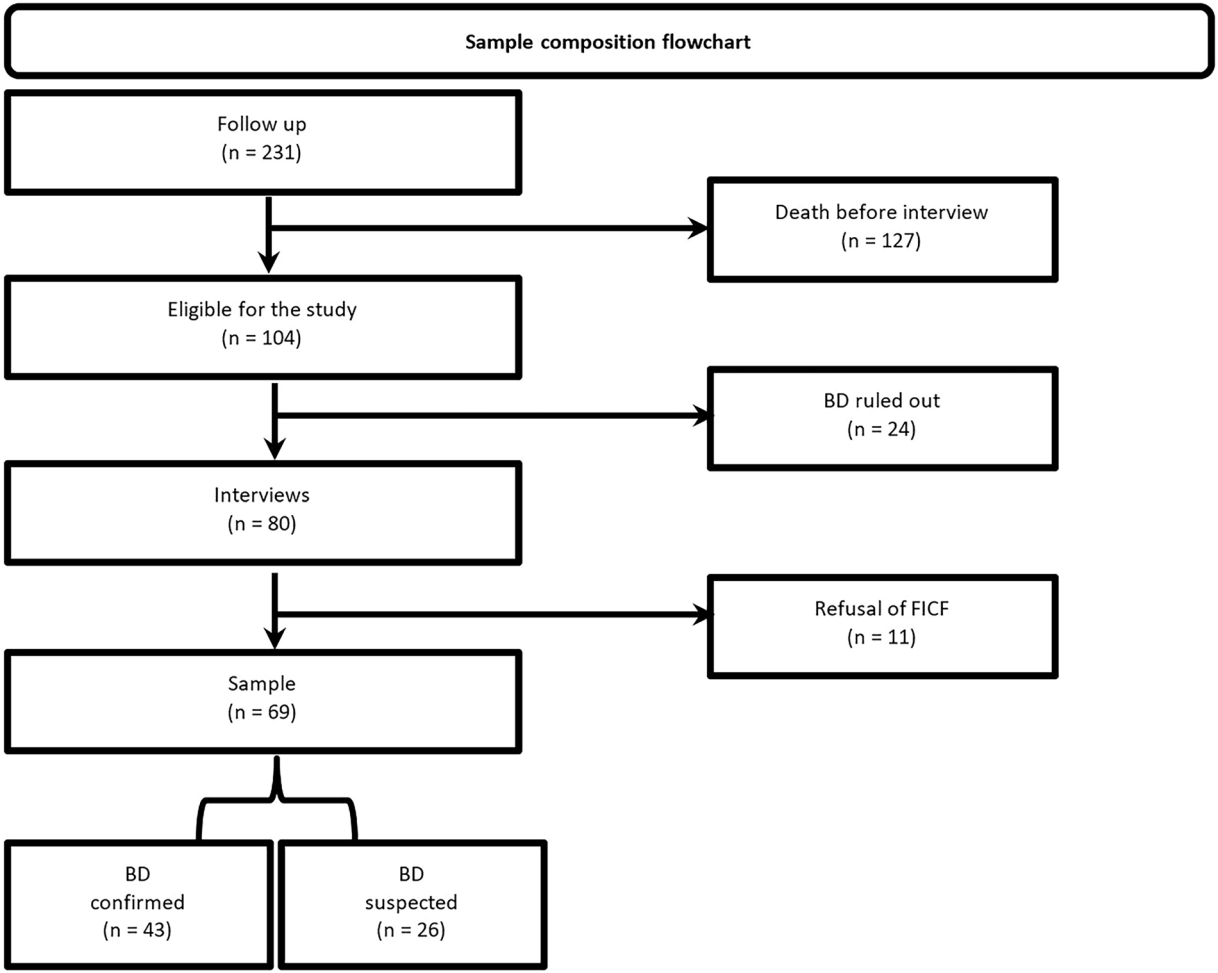
Flowchart of sample composition and distribution of outcome groups.

The sample was defined based on the monitoring of patients who met the inclusion criteria (*n* = 231). Of these, patients who died before the interview (*n* = 127), those whose BD diagnosis was ruled out (*n* = 24), and patients whose guardians did not consent to signing the informed consent form (*n* = 11) were excluded, totaling 69 participants (Figure 2).

The study included 69 patients, with a median age of 52.0 [42.5; 67.8] years, with a higher prevalence of females (59%), and brown people (62%). The results also showed that 65% of the sample consisted of patients who lived in an inland city of the state and half had elementary education as their highest level of education. Regarding occupation, 34% were retired and the majority of participants (56%) lived on the minimum wage (Table 1).

**Table 1:** Sociodemographic and clinical characteristics of confirmed and suspected cases of brain death – Aracaju, SE, Brazil, 2024.

Characteristics	N = 69
**Age**	
Mean (Standard Deviation)	53.49 (17.29)
Median [25%;75%]	52.0 [42.0;68.0]
**Sex, n/N (%)**	
Female	41/69 (59%)
Male	28/69 (41%)
**Race, n/N (%)**	
White	16/64 (25%)
Black	9/64 (14%)
Brown	39/64 (61%)
**Marital status, n/N (%)**	
Married	19/56 (34%)
Single	23/56 (41%)
Common law marriage	11/56 (20%)
Widower	3/56 (5%)
**Level of education, n/N (%)**	
Elementary education	29/59 (49%)
High school	21/59 (36%)
Higher education	3/59 (5%)
Never studied	6/59 (10%)
**Occupation, n/N (%)**	
Retired	23/69 (33%)
Self-employed	17/69 (25%)
Private company employee	11/69 (16%)
Civil servant	5/69 (7%)
Others	13/69 (19%)
**Income, n/N (%)**	
Two to three minimum salaries	12/69 (17%)
Did not declare	19/69 (28%)
One salary	38/69 (55%)
**Institution of collection, n/N (%)**	
H2	3/69 (4%)
H4	1/69 (1%)
H3	6/69 (9%)
H1	59/69 (86%)
**Inpatient unit, n/N (%)**	
Yellow	3/69 (4%)
ICU	47/69 (68%)
Red	19/69 (28%)
**Lifestyle, n/N (%)**	
Alcoholism	13/61 (21%)
Sedentary lifestyle	31/61 (51%)
Smoking	12/61 (20%)
Denies	3/61 (5%)
Others	2/61 (3%)
**SAH, n/N (%)**	
No	14/47 (30%)
Yes	33/47 (70%)
**DM, n/N (%)**	
No	32/47 (68%)
Yes	15/47 (32%)
**Cancer, n/N (%)**	
No	44/47 (94%)
Yes	3/47 (6%)
**Cardiovascular, n / N (%)**	
No	41/47 (87%)
Yes	6/47 (13%)

Legend: n – Absolute frequency; N – Valid data; % – Percentage; SAH – Systemic Arterial Hypertension; DM – Diabetes Mellitus; ICU – Intensive Care Unit; H1 – Emergency Hospital; H2 – Philanthropic Hospital; H3 – University Hospital; H4 –General Hospital.

Data on clinical conditions showed Systemic Arterial Hypertension (SAH) as the most prevalent comorbidity (70%). Diabetes Mellitus (DM) was observed in 32% and a sedentary lifestyle was a condition present in 51% of the sample. H1 accounted for 86% of patients, with the ICU being the most prevalent hospitalization unit, with 69% of hospitalized individuals.

**Figure 3:**
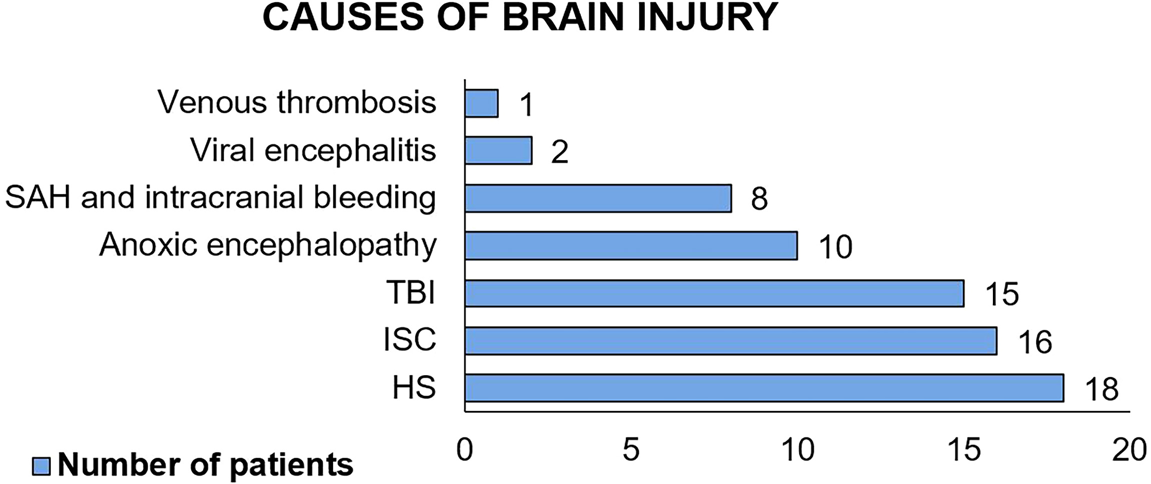
Cause of brain injury in suspected or confirmed brain death.

Regarding the cause of the brain injury (Figure 3), Hemorrhagic Stroke (HS), Ischemic Stroke (ISC) and TBI account for 18, 16 and 15 cases respectively.

Regarding the factors associated with the diagnosis of BD (Table 2), age presented a Prevalence Ratio (PR) of 0.96 (95% CI: 0.92–1.00; *p* = 0.044). The cancer variable showed a strong association with the non-confirmation of the diagnosis of BD (PR 0.18; 95% CI: 0.04–0.73; *p* = 0.022), and multivariate PR 0.20 (95% CI: 0.04–0.78; *p* = 0.027). In contrast, based on the analysis of the variable place of residence, it was observed that in all models it was significantly associated with confirmation of diagnosis. For the adjusted model, a PR of 5.49 was obtained (95% CI: 1.32–32.0; *p* = 0.031).

**Table 2:** Factors associated with the diagnosis of BD based on crude, adjusted, and multivariate models – Aracaju, SE, Brazil, 2024.

Characteristics	Crude		Adjusted		Multivariate	
	PR^1^ (95% CI)^2^	p value	PR (CI 95%)	p value	PR (CI 95%)	p value
**Age**	0.97 (0.94–1.00)	0.055	0.96 (0.92–1.00)	0.066	0.96 (0.92–1.00)	0.044
**Place of Residence**						
Other cities	–		–		–	
Greater Aracaju	5.38 (1.48–19.6)	0.011	5.49 (1.32–32.0)	0.031	5.26 (1.37–27.2)	0.025
**Cancer**						
No	–		–		–	
Yes	0.49 (0.17–1.45)	0.199	0.18 (0.04–0.73)	0.022	0.20 (0.04–0.78)	0.027

Source: Prepared by the author (2024).

Legend: ^1^PR = Prevalence ratio. ^2^CI = 95% Confidence Interval.

## DISCUSSION

This study identified a majority of individuals with a median age of 52 years, predominantly female, brown skin color, single marital status, and level of education up to elementary school (Table 1). These results are in line with data found in a survey carried out with 60 potential organ donors in Natal/RN^(13)^.

However, other results highlight a higher prevalence of male individuals, such as the North American study that found a male predominance (56.47%) among cases of BD^(2)^. Furthermore, another study showed a higher prevalence of men (61.99%) compared to women (38.01%)^(14)^.

The findings of the main causes of BD in this study reinforce other results on the global scenario. In Türkiye, between 2020 and 2023, the main causes of BD were stroke and HS, representing 45.6% and 47% of cases. TBI, the second cause, accounted for 20.7% and 21% of cases in the years studied, while ischemic stroke was the third, with a prevalence of 17.8% and 18%, respectively^(14)^.

In Brazil, the scenario is similar to other countries. Stroke and TBI are among the main causes, accounting for more than 90% of cases^(15)^. Other evidence showed subarachnoid hemorrhage in 58.2% of cases, while ischemic stroke in 7.1% and TBI in 19.4% of diagnoses^(16)^.

When comparing the results of this study with those found in the last study carried out in Sergipe, a change in the prevalence of neurological causes of BD can be seen. In the State, TBI was once the main cause of BD, representing 68.3% of cases, followed by stroke with 28.6%, while primary tumors of the central nervous system, 3.1% of the sample^(17)^.

In Brazil, although TBI is among the main causes, in 2023, according to data from the Brazilian Association of Organ Transplants (ABTO), BD became more prevalent in patients with stroke (52% of causes), followed by TBI (31% of causes), and post-cardiorespiratory arrest anoxia^(18)^. The observed transition indicates that the causes may be conditioned by the epidemiological profile, lifestyle habits, and population aging, the latter associated with cerebrovascular diseases, as they are more prevalent in older patients^(19)^.

Factors associated with the diagnosis of BD (Table 2) indicate that older patients had a 4% lower chance of diagnostic confirmation. In contrast to these findings, a similar study found a mean age of 48 years among patients with confirmed BD, with at least 10% of cases involving individuals under 18 years of age^(2)^.

This association between older age and lower probability of confirmation may result from the high prevalence of TBI in younger individuals, since this trauma constitutes the second main etiology of BD^(18)^. The greater frequency of this cause in young patients directly impacts the average age of the affected population, reinforcing the predominant age profile among confirmed cases.

Furthermore, older patients tend to be less tolerant of two pathophysiological events frequently observed in BD: hemodynamic instability and intracranial hypertension (ICH). Physiological changes related to aging, such as sarcopenia, a condition prevalent with advancing age^(20)^, worsen the prognosis of TBI victims. Loss of muscle mass contributes to the systemic and cerebral hypercatabolic state resulting from the stress response, which intensifies neurological injury and worsens clinical outcome^(21)^.

Cerebral edema, the main consequence of ICH, tends to worsen in the face of other clinical factors and comorbidities common in this age group, such as hypernatremia and glycemic changes resulting from DM. These factors increase the risk of cerebral edema and diffuse axonal injury^(22)^. In this situation, the association of comorbidities and pathophysiological changes compromises the clinical stability of older patients, reducing their ability to withstand confirmatory tests for BD, leading to CRA before the completion of the diagnostic protocol^(23)^.

The identification of neoplasia during the investigation of BD has been shown to reduce the chances of diagnostic confirmation by up to 92%. Although little explored in the literature, the relationship between cancer and BD has been considered in studies where cancer patients undergoing palliative care may receive less interventionist management, which may delay or even make the completion of the diagnostic protocol unfeasible. It is important to highlight that cancer patients and those with BD generally follow different clinical guidelines, which can directly impact the diagnosis of neurological injury^(24)^.

The coexistence of these two diagnoses constitutes an absolute contraindication for organ donation. In clinical practice, this limitation reduces the commitment of medical teams to carry out all stages of the BD protocol, despite the patient and their family members having the right to obtain a definitive diagnosis^(6)^.

Additionally, reports of emotional overload and intense psychological suffering are frequent, both on the part of family members and the patients themselves. Given the severity of the condition and the possibility that the patient may not tolerate all stages of the clinical and imaging tests, in many situations the team chooses to protect them from this process, prioritizing comfort measures and choosing therapeutic limitation as the main approach with family members^(25)^.

Region of residence revealed a significant association with confirmed BD. Specifically, residents of Greater Aracaju had a 449% increase in the chances of having BD confirmed, compared to those residing in other locations. This finding indicates disparities in access and diagnostic effectiveness within regions.

This result raises questions about the effectiveness of one of the basic principles of the Health Care Network, the integrality of health care. The observed disproportion may be related to failures in coordination between services in the inland cities and the capital, which hinders the performance of additional tests in a timely manner and to properly manage BD protocols. The fragmentation of the network and the concentration of resources in urban centers negatively impact equity in access to specialized services, which may reflect the difficulties encountered outside Greater Aracaju^(26)^.

Otherwise, the literature points to an association between the patient’s place of residence and the prevalence of BD cases. In Campo Grande/MS, for example, a higher prevalence was found in the capital, with 39 cases (69.7%), compared to 17 cases (30.3%) in the inland area^(27)^. This difference can be explained by the concentration of better health centers in the capitals, with better infrastructure, more technology, sophisticated equipment and specialized professionals, factors that increase the chances of successful diagnoses^(28)^.

In Sergipe, the only public referral center for managing BD cases is the public emergency referral hospital in the capital, which has backup beds and a team working 24 hours a day to actively search for cases, implement protocols, and enable organ donation policies. This structure promotes greater agility in diagnosis and case management, which may have influenced the results of this association.

The loss of potential participants before signing the FICF due to death from hemodynamic instability, as well as the lack of knowledge of family members about BD, hindered the inclusion of participants in this study. However, the study fills important gaps in the literature, providing a comprehensive overview of the factors associated with the diagnosis of BD in Sergipe. Through the results, it is possible to propose improvements in the diagnostic processes in the State, as well as optimize public health policies aimed at organ donation.

In this context, it is essential that nurses play the role of leader and educator, in collaboration with the multidisciplinary team, to transform this reality, while raising awareness within the population, especially among patients’ family members, about the meaning of BD and its importance for public organ donation policies.

Continuing education for nursing professionals has proven to be an effective way to improve the quality of care provided and, in the context of BD, this strategy improves understanding of the diagnostic process, clinical approach and early monitoring of possible cases, in addition to contributing to the effectiveness of organ donation policies^(29)^. It reduces the frequent delay in opening protocols due to lack of knowledge in the proper management of patients with suspected BD, either due to uncontrolled temperatures, hemodynamic instability, or blood gas parameters that prevent opening the protocol, resulting in greater agility and speed in diagnosis^(30)^.

## CONCLUSION

Confirmation of the diagnosis of BD is directly influenced by the proximity of patients’ homes to well-structured health centers, which favors the agility and accuracy of the process. However, in remote regions with limited access to specialists, it is necessary to adopt innovative strategies to ensure diagnostic efficiency, minimizing inequalities in care.

Among these strategies, the creation of institutional protocols with checklists and evidence-based flows, with the aim of promoting standardization, accuracy and agility in diagnosis is highlighted. Such measures can reduce errors and optimize BD confirmation time, even in units with limited resources. Additionally, the use of telehealth as an innovative tool can mitigate the impacts of the shortage of specialists by enabling remote consultations with neurologists and intensivists, allowing the validation of clinical criteria, diagnostic procedures, and analysis of exams, such as CT scans.

Another essential aspect is the continuous investment in training for the nursing team, aimed at early identification of cases and monitoring signs of clinical instability. The integration of well-defined protocols, telemedicine consultations, and professional training significantly contributes to faster, safer, and more equitable diagnosis, especially in contexts of structural vulnerability.

## Data Availability

The complete dataset underpinning the results of this study has been made publicly available within the article itself.

## References

[1] Milliken A, Uveges MCE (2020). Brain death: history, updates, and implications for
nurses. Am J Nurs.

[2] Seifi A, Lacci JV, Godoy DA (2020). Incidence of brain death in the United States. Clin Neurol Neurosurg.

[3] Starr R, Tadi P, Pfleghaar N (2024). Brain death [Internet].

[4] Souza DH, Costa LC, Barbosa TP, Chieratto CLD, Olivares NM, Ornelas J (2021). Determinação de morte encefálica, captação e doação de órgãos e
tecidos em um hospital de ensino. Cuid Enferm.

[5] Greer DM, Kirschen MP, Lewis A, Gronseth GS, Rae-Grant A, Ashwal S (2023). Pediatric and Adult Brain Death/Death by Neurologic Criteria
Consensus Guideline: Report of the AAN Guidelines Subcommittee, AAP, CNS,
and SCCM. Neurology.

[6] Brasil (2017). Federal de Medicina. Resolução n^o^ 2173, de 23 de novembro de
2017. Define os critérios do diagnóstico de morte encefálica.

[7] Greer DM, Shemie SD, Lewis A, Torrance S, Varelas P, Goldenberg FD (2020). Determination of brain death/death by neurologic criteria: The
World Brain Death Project. JAMA.

[8] Wahlster S, Wijdicks EFM, Patel PV, Greer DM, Hemphill JC, Carone M (2015). Brain death declaration. Neurology.

[9] Alves MP, Rodrigues FS, Da Cunha KS, Higashi GDC, Nascimento ERP, Erdmann AL (2019). Processo de morte encefálica: significado para enfermeiros de uma
unidade de terapia intensiva. Rev Baiana Enferm.

[10] Da Silva FA, Vieira EMS, Da Silva ACR (2024). Morte encefálica e equipe de enfermagem: desafios e saberes – uma
revisão de literatura. Rev ft.

[11] Flores CML, Silva RM, Tamiozzo J, Centenaro APFC, Silva DMGV, Zamberlan C (2023). Care for potential brain-dead organ donors in an adult emergency
room: a convergent care perspective. Texto Context -Enferm.

[12] Sergipe. Central de Transplantes (2023). Relatório anual de atividades.

[13] Freire ILS, De Vasconcelos QLDAQ, Araújo RO, Pinto JTJM, Torres GV (2013). Characterization of the potential donors of organs and tissues
for transplantation. J Nurs UFPE/Rev Enferm UFPE.

[14] Sahin M, Altinay M, Cinar AS, Yavuz H (2023). Retrospective analysis of patients diagnosed with brain death in
Our Hospital in the Last 15 years. Sisli Etfal Hastan Tip Bul.

[15] Sindeaux ACA, Nascimento AMV, Campos JRE, Campos JBR, Barros AB, Luz DCRP (2021). Cuidados de enfermagem dispensados ao potencial doador de órgãos
em morte encefálica: uma revisão integrativa. Nursing (São Paulo).

[16] Souza DH, Costa LC, Barbosa TP, Chieratto CLD, Olivares NM, Ornelas J (2021). Determinação de morte encefálica, captação e doação de órgãos e
tecidos em um hospital de ensino. CuidArte, Enferm.

[17] Nogueira EC, Pereira CU (2007). Potencial para obtenção de órgãos em um hospital de urgência de
Sergipe. Brazilian J Transplant.

[18] Associação Brasileira de Transplante de Órgãos (2023). Registro Brasileiro de Transplantes: dimensionamento dos transplantes no
Brasil e em cada estado (2016–2023).

[19] Izzo C, Carrizzo A, Alfano A, Virtuoso N, Capunzo M, Calabrese M (2018). The impact of aging on cardio and cerebrovascular
diseases. Int J Mol Sci.

[20] Cho MR, Lee S, Song SK (2022). A review of sarcopenia pathophysiology, diagnosis, treatment and
future direction. J Korean Med Sci.

[21] Carney N, Totten AM, O’Reilly C, Ullman JS, Hawryluk GWJ, Bell MJ (2017). Guidelines for the Management of Severe Traumatic Brain Injury,
Fouth edition. Neurosurgery.

[22] Carvalho AS (2018). Associação de hipernatremia com o prognóstico e a mortalidade de
pacientes com traumatismo cranioencefálico grave em um hospital terciário
brasileiro [dissertação].

[23] Ali SMM, El-Bouri W, Mokhtarudin MJM (2022). Proceedings of the 2022 IEEE-EMBS Conference on Biomedical Engineering
and Sciences (IECBES); 2022; Kuala Lumpur, Malaysia.

[24] Mota RV, Coelho NS, Studart RMB, Passos MMVS, Henrique TLS, Sousa MAO (2024). Perfil clínico e epidemiológico de pacientes com diagnóstico de
morte encefálica atendidos na emergência. Rev Contemp.

[25] Parvizi M, Ay S (2024). The assessment of care burden and influencing factors on family
caregivers for cancer patients. J Clin Nurs.

[26] Viana d’Ávila AL, Bousquat A, Melo GA, Negri Fo A, Medina MG (2018). Regionalização e Redes de Saúde. Cien Saude Colet.

[27] Pogodin GF, Souza MC, Pompeo CM, Ferreira MA, Hildebrand CR, Ivo ML (2023). Caracterização epidemiológica e causas da não doação por
potenciais doadores de órgãos em morte encefálica. Rev Enferm UERJ.

[28] Fonseca BS, Souza VS, Batista TOF, Silva GM, Spigolon DN, Derenzo N (2021). Strategies for hemodynamic maintenance of potential brain-dead
donor: integrative review. Einstein (Sao Paulo).

[29] Pavan AJ, Dallagnol P, Narzetti RA, Levinsk DJ, Brustolin AM, Freitas TLL (2025). Atuação do enfermeiro no processo de doação de órgãos em morte
encefálica. Saúde Colet (Barueri).

[30] Pimentel RRS, Dos Santos MJ, Martins MS, Brito AN, Hidalgo BRG, Gonçalves No C (2022). Understanding of Brazilian Nursing Assistants and Technicians of
Brain Death. Transplant Proc.

