# Supplementary material for: Factors associated with the diagnosis of Brain Death in hospitals in the State of Sergipe
Source: Rev Esc Enferm USP. 2025 Oct 27;59:e20250014. doi: 10.1590/1980-220X-REEUSP-2025-0014en (PMC12594460; doi:10.1590/1980-220X-REEUSP-2025-0014en)
Supplement: Translation

# Translation: Fatores associados ao diagnóstico de Morte Encefálica em hospitais no Estado de Sergipe

**DOI:** 10.1590/1980-220X-REEUSP-2025-0014pt

**Authors:** José Lucas dos Santos, Gabriella Santos Cisneiros, Jussiely Cunha Oliveira, Eduesley Santana Santos, Fernanda Gomes de Magalhães Soares Pinheiro

**Affiliations:** 1 Universidade Federal de Sergipe, Programa de Pós-Graduação em Enfermagem, Aracaju, SE, Brasil.; 2 Universidade Federal de Sergipe, Lagarto, SE, Brasil.; 3 Universidade Federal de Sergipe, Departamento de Enfermagem, Lagarto, SE, Brasil.; 4 Universidade Federal de Sergipe, Departamento de Enfermagem, Aracaju, SE, Brasil.

**Keywords:** Adulto, Morte Encefálica, Epidemiologia, Diagnóstico

## Abstract

**Objetivo:**

Analisar os fatores associados ao diagnóstico de morte encefálica (ME) em hospitais do Estado de Sergipe.

**Método:**

Estudo transversal realizado entre 2023 e 2024 em quatro hospitais de Sergipe. Participaram maiores de 18 anos, de ambos os sexos, três pontos na Escala de Coma de Glasgow, lesão neurológica comprovada por tomografia de crânio e ausência de pelo menos dois reflexos de tronco.

**Resultados:**

O estudo incluiu 69 participantes e foi observada maior prevalência de indivíduos provenientes do interior (65%), com idade mediana de 52 anos (42,0–68,0), do sexo feminino (59%) e de etnia parda (62%). As principais causas neurológicas foram acidente vascular cerebral e traumatismo cranioencefálico. Indivíduos em faixa etária mais avançada e indivíduos com câncer tiverem menos chances de confirmar ME. Já a variável local de residência esteve significativamente associada ao diagnóstico.

**Conclusão:**

Recomenda-se a integração entre protocolos institucionais bem definidos, consultorias por telessaúde e qualificação profissional como estratégia que pode levar a um diagnóstico mais **ágil**, seguro e equitativo, em contextos de vulnerabilidade estrutural.

## INTRODUÇÃO

O conceito de morte biológica foi reformulado em 1981 com base na Lei de Determinação Uniforme da Morte (UDDA). Essa legislação definiu a morte como um evento caracterizado pela cessação irreversível das funções circulatórias e respiratórias (critérios cardiopulmonares) ou pela cessação irreversível de todas as funções cerebrais, incluindo as do tronco encefálico^(1)^.

A partir da UDDA, o Brasil e outros países adotaram o conceito de Morte Encefálica (ME). Assim, a morte cerebral é caracterizada pela cessação irreversível das funções dos hemisférios cerebrais e do tronco encefálico, decorrente do comprometimento da circulação cerebral, secundário a uma lesão conhecida e irreversível^(2)^.

O mecanismo da ME parte de uma descompensação na Pressão de Perfusão Cerebral (PPC), que é o resultado da diferença entre a Pressão Arterial Média (PAM) e a Pressão Intracraniana (PIC). O aumento da PIC reduz a PPC que, consequentemente, compromete a circulação em todo o cérebro. Essas disfunções pressóricas provocam danos cerebrais e favorecem a instalação de edema progressivo, devido à oxigenação inadequada. Esse processo agrava ainda mais a PIC, podendo culminar em herniação, interrupção do fluxo sanguíneo cerebral e necrose. Nas lesões anóxicas, a hipóxia estimula a liberação de substâncias citotóxicas e o desenvolvimento de edema cerebral^(3)^.

As principais causas de ME estão relacionadas a eventos que afetam o cérebro, induzindo edema progressivo e subsequente lesão irreversível. Entre as etiologias mais comuns, destacam-se as doenças cerebrovasculares, o Traumatismo Cranioencefálico (TCE), a anóxia pós-Parada Cardiorrespiratória (PCR), as infecções e os tumores do sistema nervoso central^(4)^.

Na maioria dos países, o diagnóstico da ME baseia-se na avaliação neurológica das funções de todo o cérebro e do tronco encefálico, associada à ausência de movimentos respiratórios. Nos Estados Unidos, o diagnóstico de ME é orientado por *guidelines* e protocolos estabelecidos por organizações médicas renomadas^(5)^.

Já no Brasil, a Resolução do Conselho Federal de Medicina (CFM) nº 2.173/2017 foi formulada a fim de definir os critérios para o diagnóstico da morte neurológica, conferindo rigor ao processo. A confirmação dessa lesão cerebral no país baseia-se na realização de dois exames clínicos com intervalo mínimo de uma hora para pacientes maiores de dois anos, na realização de um teste de apneia e um exame complementar^(6)^; porém, divergências permeiam sua condução em todo o mundo. Diante disso, especialistas globais consentiram com a formulação de declarações que direcionam os critérios neurológicos da ME, visando uniformizá-lo o máximo possível e garantir maior criteriosidade ao processo^(7)^.

A ausência de protocolos institucionais para o diagnóstico de ME, a falta de leitos de UTI, a indisponibilidade de recursos tecnológicos, a escassez de profissionais experientes e o conhecimento técnico profissional incipiente estão fortemente associados às dificuldades no diagnóstico de ME. Tais fatores refletem disparidades na compreensão e no diagnóstico dessa condição, contribuindo para lacunas na educação médica, na disponibilidade de recursos humanos e na utilização de tecnologias adequadas^(8)^.

Nesse contexto, o enfermeiro desempenha papel essencial na prevenção de atrasos e na viabilização do diagnóstico de ME. A atuação contínua desse profissional na UTI possibilita a identificação precoce da piora neurológica, a comunicação eficaz com a equipe interdisciplinar e a preparação adequada para os exames necessários ao diagnóstico^(9)^.

Embora o enfermeiro seja reconhecido no gerenciamento burocrático da assistência em ME, sua eficácia na assistência e na gestão da equipe depende do desenvolvimento de habilidades técnicas e não técnicas, aprimoradas por meio da experiência e da educação continuada^(10)^. A ausência desse aprimoramento compromete sua autoconfiança e a qualidade da assistência, podendo levar a atrasos ou falhas no diagnóstico da morte encefálica^(11)^.

Dessa forma, a atuação proativa do enfermeiro, por meio da educação continuada da equipe, aprimora os fluxos institucionais. Esse profissional também exerce um papel estratégico na orientação dos familiares, esclarecendo a irreversibilidade do quadro e reduzindo resistências que possam comprometer o diagnóstico e a doação de órgãos. A participação do enfermeiro na construção de protocolos institucionais padroniza condutas, tornando o fluxo mais ágil e assertivo na confirmação da ME^(11)^.

Este estudo preenche uma lacuna importante na literatura e oferece uma base para estudos futuros na área, evidenciando as dificuldades estruturais e educacionais que podem impactar na conclusão dos protocolos. Para este estudo levantou-se como questão principal “Quais fatores clínicos, sociodemográficos e estruturais estão associados ao diagnóstico de ME em hospitais do Estado de Sergipe”?

Assim, o estudo baseia-se na hipótese de que residir no interior pode estar associado à não confirmação do diagnóstico de ME, devido a múltiplos fatores que dificultam a conclusão dos protocolos. Entre eles, destacam-se as limitações na estrutura tecnológica, a indisponibilidade de exames de imagem complementares, a heterogeneidade institucional em termos de complexidade assistencial, a escassez de recursos humanos especializados e os déficits na capacitação profissional. Esses fatores, em conjunto, podem contribuir para o aumento do tempo necessário para a confirmação do diagnóstico.

O objetivo deste estudo foi analisar os fatores associados ao diagnóstico de ME em hospitais no Estado de Sergipe.

## MÉTODO

### Desenho do Estudo

Trata-se de um estudo transversal, guiado pelas recomendações do *Strengthening the Reporting of Observational Studies in Epidemiology* (STROBE).

### Local

Este estudo foi conduzido em quatro hospitais sergipanos. Na capital Aracaju foram dois, sendo um hospital de urgência (H1) e um hospital filantrópico (H2). No interior, um hospital universitário (H3), situado na cidade de Lagarto, e um hospital geral (H4), situado na cidade de Itabaiana. A seleção dessas unidades hospitalares baseou-se na Resolução do CFM 2.173/2017, que exige a realização de exame de neuroimagem para identificação da causa neurológica em pacientes elegíveis para o protocolo de ME^(10)^. Portanto, todos os hospitais participantes dispõem de serviço de neuroimagem 24 horas e serviço de urgência e emergência.

### População e Critérios de Seleção

Foram incluídos participantes maiores de 18 anos, de ambos os sexos, que apresentaram um escore de três pontos na ECG, com lesão neurológica comprovada por meio de tomografia de crânio realizada previamente à determinação de morte encefálica, e que apresentavam ausência de dois dos seguintes reflexos: tosse, pupilar e respiratório. Foram excluídos os participantes que tiveram o diagnóstico de ME descartado, que estavam em uso de sedação e os que foram a óbito antes da realização da tomografia de crânio.

### Cálculo do Tamanho da Amostra

Foi utilizado como recorte temporal o ano de 2022 e os 212 protocolos iniciados no Estado e notificados à Organização de Procura de Órgãos e Tecidos para Transplantes^(12)^. Assumindo uma população finita, com um nível de significância de 5%, margem de erro de 10% e uma prevalência de 50%, o tamanho amostral calculado foi de no mínimo 67 participantes, baseado na seguinte fórmula:

$$n=\frac{NZ_{\frac{\alpha }{2}}^{2}p(1-p)}{e^{2}(N-1)+Z_{\frac{\alpha }{2}}^{2}p(1-p)}$$

Onde *n* é o tamanho da amostra, *N* é o tamanho da população, *p* é a proporção esperada, *e* é a margem de erro e $Z_{\frac{\alpha }{2}}^{2}$ é o quadrado do escore da distribuição normal associado ao nível de significância *α*.

$$n=\frac{212\times {\left(1,965\right)}^{2}\times 0,5\times (1-0,5)}{{\left(0,10\right)}^{2}(212-1)+{\left(1,965\right)}^{2}\times 0,5\times (1-0,5))}\approx 67$$

### Coleta de Dados

O período de coleta de dados foi de agosto de 2023 a agosto de 2024 e ocorreu por meio de entrevistas com os familiares e responsáveis legais dos participantes, análise de registros e prontuário do paciente. O instrumento de coleta foi estruturado e elaborado pelo autor principal, com base em formulários pré-existentes da Organização de Procura de Órgãos, e dispunha de perguntas abertas e fechadas.

A equipe responsável pela coleta de dados foi composta por estudantes de graduação do curso de enfermagem, pós-graduação e profissionais voluntários. Todos os integrantes receberam treinamento teórico sobre a temática, os objetivos do estudo, os critérios de elegibilidade dos participantes e o preenchimento do instrumento de coleta de dados. Para a calibração dos pesquisadores, foram realizadas coletas teste nos quatro hospitais, com o objetivo de avaliar a padronização das coletas e eficácia do instrumento na obtenção dos dados necessários para o estudo.

As entrevistas permitiram a obtenção de informações sobre variáveis sociodemográficas, como raça, estado civil, naturalidade, escolaridade, número de filhos, profissão e renda, além do histórico de doenças prévias, quando esses dados não estavam registrados nos prontuários dos participantes. As etapas da coleta de dados estão detalhadas na Figura 1.

**Figura 1:** Sistemática da coleta de dados.

Os participantes foram selecionados por meio de busca ativa nas unidades de emergência e Unidades de Terapia Intensiva das instituições. Após a verificação dos critérios de inclusão, o Termo de Consentimento Livre e Esclarecido foi aplicado ao familiar ou responsável legal por meio de entrevista.

### Análise E Tratamento dos Dados

A análise estatística foi realizada utilizando medidas de tendência central (média e mediana), dispersão (desvio padrão e intervalo interquartil) e distribuição (frequência absoluta e percentual).

A associação entre variáveis categóricas foi avaliada por meio do teste do Qui-quadrado, sendo aplicado o teste exato de Fisher quando apropriado. Para comparação das medianas entre duas ou mais amostras independentes, foram utilizados os testes de *Wilcoxon-Mann-Whitney*, *Kruskal-Wallis* e para identificar especificamente quais grupos diferiam entre si foi utilizado o teste de *Dunn,* aplicado como *post hoc*.

A regressão logística foi empregada para modelar a relação entre variáveis preditoras e desfechos binários, utilizando modelos brutos (com apenas a variável de interesse), ajustados e multivariados, considerando apenas variáveis significativas no modelo final. Foram adotados como critérios de seleção a significância inferior a 0,2, a ausência de fenômenos de separação e a ausência de multicolinearidade. O método de seleção de variáveis utilizado foi o *backward*.

As análises estatísticas foram conduzidas no ambiente de programação R (versão 4.4.0) (R CORE TEAM, 2023), adotando um nível de significância de 5%.

### Aspectos Éticos

Esse estudo foi aprovado pelo Comitê de Ética em Pesquisa (CEP) da Universidade Federal de Sergipe em 21 de junho de 2023 sob o número CAAE: 68731823.3.0000.5546 e número do parecer 6.132.287.

## RESULTADOS

A amostra foi definida a partir do acompanhamento de pacientes que atendiam aos critérios de inclusão (*n* = 231). Desses, foram excluídos os pacientes que foram a óbito antes da entrevista (*n* = 127), que tiveram o diagnóstico de ME descartado (*n* = 24) e os pacientes cujos responsáveis não consentiram com a assinatura do TCLE (*n* = 11), totalizando 69 participantes (Figura 2).

**Figura 2:** Fluxograma de composição da amostra e distribuição dos grupos de desfechos.

O estudo incluiu 69 pacientes, sendo a mediana de idade de 52,0 [42,5; 67,8] anos, com maior prevalência do sexo feminino (59%) e etnia parda (62%). Os resultados mostraram ainda que 65% da amostra foi composta por pacientes que residiam no interior do Estado e metade tinha o ensino fundamental como maior escolaridade. Quanto à ocupação, 34% eram aposentados e a maioria dos participantes (56%) vivia com um salário-mínimo (Tabela 1).

**Tabela 1:** Características sociodemográficas e clínicas de casos confirmados e suspeitos de morte encefálica – Aracaju, SE, Brasil, 2024.

Características	N = 69
**Idade**	
Média (Desvio Padrão)	53,49 (17,29)
Mediana [25%;75%]	52,0 [42,0;68,0]
**Sexo, n/N (%)**	
Feminino	41/69 (59%)
Masculino	28/69 (41%)
**Raça, n/N (%)**	
Branca	16/64 (25%)
Negra	9/64 (14%)
Parda	39/64 (61%)
**Estado civil, n/N (%)**	
Casado	19/56 (34%)
Solteiro	23/56 (41%)
União estável	11/56 (20%)
Viúvo	3/56 (5%)
**Escolaridade, n/N (%)**	
Ensino fundamental	29/59 (49%)
Ensino médio	21/59 (36%)
Ensino superior	3/59 (5%)
Nunca estudou	6/59 (10%)
**Ocupação, n/N (%)**	
Aposentado	23/69 (33%)
Autônomo	17/69 (25%)
Funcionário privado	11/69 (16%)
Funcionário público	5/69 (7%)
Outros	13/69 (19%)
**Renda, n/N (%)**	
Dois a três salários	12/69 (17%)
Não declarou	19/69 (28%)
Um salário	38/69 (55%)
**Instituição coleta, n/N (%)**	
H2	3/69 (4%)
H4	1/69 (1%)
H3	6/69 (9%)
H1	59/69 (86%)
**Unidade de internação, n/N (%)**	
Amarela	3/69 (4%)
UTI	47/69 (68%)
Vermelha	19/69 (28%)
**Estilo de vida, n/N (%)**	
Etilismo	13/61 (21%)
Sedentarismo	31/61 (51%)
Tabagismo	12/61 (20%)
Nega	3/61 (5%)
Outros	2/61 (3%)
**HAS, n/N (%)**	
Não	14/47 (30%)
Sim	33/47 (70%)
**DM, n/N (%)**	
Não	32/47 (68%)
Sim	15/47 (32%)
**Câncer, n/N (%)**	
Não	44/47 (94%)
Sim	3/47 (6%)
**Cardiovascular, n/N (%)**	
Não	41/47 (87%)
Sim	6/ 47 (13%)

Legenda: n – Frequência absoluta; N – Dados válidos; % – Percentual; HAS – Hipertensão Arterial Sistêmica; DM – Diabetes Mellitus; UTI – Unidade de terapia Intensiva; H1 – Hospital de Urgência; H2 – Hospital Filantrópico; H3 – Hospital Universitário; H4 – Hospital Geral.

Os dados sobre as condições clínicas mostraram Hipertensão Arterial Sistêmica (HAS) como a comorbidade de maior prevalência (70%). O Diabetes Mellitus (DM) foi observado em 32% e o sedentarismo foi uma condição presente em 51% da amostra. O H1 concentrou 86% dos pacientes, sendo a UTI a unidade de internação mais prevalente, com 69% dos indivíduos internados.

Quanto à causa da lesão encefálica (Figura 3), Acidente Vascular Cerebral Hemorrágico (AVCh), Acidente Vascular Cerebral Isquêmico (AVCi) e TCE, responsáveis por 18, 16 e 15 casos respectivamente.

**Figura 3:** Causa da lesão encefálica na suspeita ou confirmação de morte encefálica.

Quanto aos fatores associados ao diagnóstico de ME (Tabela 2), a idade apresentou Razão de Prevalência (RP) de 0,96 (IC 95%: 0,92–1,00; *p* = 0,044). Já a variável câncer apresentou forte associação com a não confirmação do diagnóstico de ME (RP 0,18; IC 95%: 0,04–0,73; *p* = 0,022) e multivariado RP 0,20 (IC 95%: 0,04–0,78; *p* = 0,027). Por outro lado, com base na análise da variável local de residência, observou-se que em todos os modelos esteve significativamente associada a confirmação do diagnóstico. Para o modelo ajustado obteve-se uma RP de 5,49 (IC 95%:1,32–32,0; *p* = 0,031).

**Tabela 2:** Fatores associados ao diagnóstico de ME baseado em modelos bruto, ajustado e multivariado – Aracaju, SE, Brasil, 2024.

Características	Bruto		Ajustado		Multivariado	
	RP^1^ (IC 95%)^2^	Valor p	RP (IC 95%)	Valor p	RP (IC 95%)	Valor p
**Idade**	0,97 (0,94−1,00)	0,055	0,96 (0,92–1,00)	0,066	0,96 (0,92–1,00)	0,044
**Local de Residência**						
Outras cidades	–		–		–	
Grande Aracaju	5,38 (1,48–19,6)	0,011	5,49 (1,32–32,0)	0,031	5,26 (1,37–27,2)	0,025
**Câncer**						
Não	–		–		–	
Sim	0,49 (0,17–1,45)	0,199	0,18 (0,04–0,73)	0,022	0,20 (0,04–0,78)	0,027

Fonte: Elaborado pelo autor (2024).

Legenda: ^1^RP = Razão de prevalência. ^2^IC = Intervalo com 95% de Confiança.

## DISCUSSÃO

Este estudo identificou uma maioria de indivíduos com mediana de 52 anos, predominância do sexo feminino, cor parda, estado civil solteiro e escolaridade até o ensino fundamental (Tabela 1). Esses resultados estão em consonância com os dados encontrados em uma pesquisa realizada com 60 potenciais doadores de órgãos em Natal/RN^(13)^.

Entretanto, outros resultados destacam uma maior prevalência em indivíduos do sexo masculino, como por exemplo, o estudo norte-americano que encontrou uma predominância masculina (56,47%) entre os casos de ME^(2)^. Por mais, outro estudo mostrou uma prevalência maior em homens (61,99%) em comparação com mulheres (38,01%)^(14)^.

Os achados das principais causas de ME neste estudo fortalecem outros resultados no cenário mundial. Na Turquia, entre 2020 e 2023, verificou-se que as principais causas de ME foram o AVCh e a hemorragia intracraniana, representando 45,6% e 47% dos casos. O TCE, a segunda causa, foi responsável por 20,7% e 21% dos casos nos anos estudados, enquanto o AVCi a terceira, com prevalência de 17,8% e 18%, respectivamente^(14)^.

No Brasil, o cenário é semelhante aos outros países. O AVC e o TCE estão entre as principais causas, somando mais de 90% dos casos^(15)^. Outras evidências trouxeram a hemorragia subaracnóidea em 58,2% dos casos, enquanto o AVCi 7,1% e o TCE 19,4% dos diagnósticos^(16)^.

Ao comparar os resultados deste estudo com os encontrados no último estudo realizado em Sergipe, percebe-se uma mudança na prevalência das causas neurológicas de ME. No Estado, o TCE já foi a principal causa de ME, representando 68,3% dos casos, seguido pelo AVC com 28,6%, enquanto os tumores primários do sistema nervoso central, 3,1% da amostra^(17)^.

No Brasil, apesar de o TCE estar entre as principais causas, no ano de 2023, de acordo com dados da Associação Brasileira de Transplantes de Órgãos (ABTO), a ME passou a ser mais prevalente em pacientes com AVC (52% das causas), seguidos pelo TCE (31% das causas) e pela anóxia pós-parada cardiorrespiratória^(18)^. A transição que é observada indica que as causas podem estar condicionadas ao perfil epidemiológico, hábitos de vida e o envelhecimento populacional, esse último associado a doenças cerebrovasculares, já que são mais prevalentes em pacientes de maior idade^(19)^.

Os fatores associados ao diagnóstico de ME (Tabela 2) apontam que pacientes com maior faixa etária apresentaram uma chance 4% menor de confirmação diagnóstica. Contrastando com esses achados, um estudo semelhante encontrou uma média de idade de 48 anos entre os pacientes com ME confirmada, sendo que pelo menos 10% dos casos envolveram indivíduos com menos de 18 anos^(2)^.

Esta associação entre idade avançada e menor probabilidade de confirmação pode decorrer da elevada prevalência de TCE em indivíduos mais jovens, uma vez que esse trauma constitui a segunda principal etiologia da ME^(18)^. A maior frequência dessa causa em pacientes jovens impacta diretamente na média etária da população acometida, reforçando o perfil etário predominante entre os casos confirmados.

Além disso, pacientes de maior idade tendem a tolerar menos dois eventos fisiopatológicos frequentemente observados na ME: a instabilidade hemodinâmica e a hipertensão intracraniana (HIC). As alterações fisiológicas relacionadas ao envelhecimento, como a sarcopenia, condição prevalente com o avanço da idade^(20)^, agravam o prognóstico de vítimas de TCE. A perda de massa muscular contribui para o estado hipercatabólico sistêmico e cerebral decorrente da resposta ao estresse, o que intensifica a lesão neurológica e piora o desfecho clínico^(21)^.

O edema cerebral, principal consequência da HIC, tende a se agravar diante de outros fatores clínicos e comorbidades comuns nessa faixa etária, como a hipernatremia e alterações glicêmicas decorrentes do DM. Esses fatores aumentam o risco de edema cerebral e de lesão axonal difusa^(22)^. Frente a essa situação, a associação de comorbidades e alterações fisiopatológicas compromete a estabilidade clínica de pacientes em maior faixa etária, reduzindo sua capacidade de suportar os testes confirmatórios para ME cursando para PCR antes da conclusão do protocolo diagnóstico^(23)^.

A identificação de neoplasia durante a investigação de ME demonstrou reduzir em até 92% as chances de confirmação diagnóstica. Embora pouco explorada na literatura, a relação entre câncer e ME tem sido considerada em estudos onde pacientes oncológicos, submetidos a cuidados paliativos, podem receber manejos menos intervencionistas, o que pode retardar ou até inviabilizar a conclusão do protocolo diagnóstico. Cumpre destacar que pacientes oncológicos e aqueles em ME geralmente seguem diretrizes clínicas distintas, o que pode impactar diretamente a condução do diagnóstico da lesão neurológica^(24)^.

A coexistência desses dois diagnósticos configura uma contraindicação absoluta para a doação de órgãos. Na prática clínica, essa limitação reduz o empenho das equipes médicas na realização de todas as etapas do protocolo de ME, apesar de ser um direito do paciente e de seus familiares a obtenção de um diagnóstico definitivo^(6)^.

De maneira complementar, são frequentes os relatos de sobrecarga emocional e intenso sofrimento psicológico, tanto por parte dos familiares quanto dos próprios pacientes. Frente a gravidade do quadro e da possibilidade de o paciente não tolerar todas as etapas dos exames clínicos e de imagem, em muitas situações a equipe opta por preservá-los desse processo, priorizando medidas de conforto e elegendo a limitação terapêutica como principal conduta junto aos familiares^(25)^.

A região de residência revelou uma associação significativa com a confirmação de ME. Especificamente, residentes da Grande Aracaju apresentaram um aumento de 449% nas chances de confirmação de ME, em comparação com aqueles que residem em outras localidades. Tal achado indica disparidades no acesso e na efetividade do diagnóstico entre as regiões.

Esse resultado levanta questionamentos sobre a efetividade de um dos princípios basilares da Rede de Atenção à Saúde, a integralização da assistência à saúde. A desproporção observada pode estar relacionada a falhas na articulação entre os serviços do interior e da capital, o que dificulta a realização oportuna de exames complementares e a condução adequada dos protocolos de ME. A fragmentação da rede e a concentração de recursos em centros urbanos impactam negativamente a equidade no acesso aos serviços especializados, o que pode remeter às dificuldades encontradas fora da Grande Aracaju^(26)^.

De outro modo, a literatura aponta a associação entre local de residência do paciente e a prevalência de casos de ME. Em Campo Grande/MS, por exemplo, foi constatada maior prevalência na capital, com 39 casos (69,7%), em comparação a 17 casos (30,3%) no interior^(27)^. Essa diferença pode ser justificada pela concentração de melhores centros de saúde nas capitais, com melhor infraestrutura, mais tecnologias, equipamentos sofisticados e profissionais especializados, fatores que aumentam as chances de sucesso na realização dos diagnósticos^(28)^.

Em Sergipe, o único centro público de referência para condução dos casos de ME é o hospital público de referência em urgência na capital, que dispõe de leitos de retaguarda e uma equipe que atua 24 horas na busca ativa de casos, condução dos protocolos e viabilização das políticas de doação de órgãos. Essa estrutura promove maior agilidade no diagnóstico e na condução dos casos, o que pode ter influenciado os resultados dessa associação.

A perda de potenciais participantes antes da assinatura do TCLE devido ao óbito por instabilidade hemodinâmica, assim como a falta de conhecimento dos familiares sobre ME dificultaram a inclusão de participantes neste estudo. Entretanto, o estudo preenche lacunas importantes na literatura, fornecendo uma visão abrangente sobre os fatores associados ao diagnóstico de ME em Sergipe. Por meio dos resultados, é possível propor melhorias nos processos diagnósticos no Estado, bem como otimizar as políticas públicas de saúde voltadas à doação de órgãos.

Nesse contexto, é essencial que o enfermeiro desempenhe o papel de líder e educador, em colaboração com a equipe multiprofissional, a fim de transformar essa realidade, juntamente com a conscientização da população, especialmente dos familiares dos pacientes, sobre o significado da ME e sua importância para as políticas públicas de doação de órgãos.

A Educação continuada de profissionais de enfermagem é comprovada como a maneira eficaz de melhorar a qualidade da assistência prestada e, no contexto de ME, esta estratégia melhora a compreensão do processo diagnóstico, o olhar clínico e monitoramento dos possíveis casos de forma precoce, além de contribuir para a efetividade das políticas de doação de órgãos^(29)^. Ela reduz o frequente atraso na abertura dos protocolos por desconhecimento no manejo adequado do paciente com suspeita de ME, seja por descontrole das temperaturas, instabilidade hemodinâmica ou parâmetros gasométricos impeditivos à abertura do protocolo, refletindo em maior agilidade e celeridade ao diagnóstico^(30)^

## CONCLUSÃO

A confirmação do diagnóstico de ME é diretamente influenciada pela proximidade da residência dos pacientes a centros de saúde bem estruturados, o que favorece a agilidade e a precisão do processo. Entretanto, em regiões afastadas e com acesso limitado a especialistas, torna-se necessário adotar estratégias inovadoras para garantir a eficiência diagnóstica, minimizando as desigualdades no atendimento.

Entre essas estratégias, destaca-se a criação de protocolos institucionais com *checklists* e fluxos baseados em evidências, com o objetivo de promover a padronização, a precisão e a agilidade no diagnóstico. Tais medidas podem reduzir erros e otimizar o tempo de confirmação da ME, mesmo em unidades com recursos limitados. Complementarmente, o uso da telessaúde como uma ferramenta inovadora pode mitigar os impactos da escassez de especialistas com a viabilização de consultorias remotas com neurologistas e intensivistas, possibilitando a validação dos critérios clínicos, a condução diagnóstica e a análise de exames, como tomografia.

Outro aspecto essencial é o investimento contínuo na capacitação da equipe de enfermagem, voltado à identificação precoce dos casos e ao monitoramento dos sinais de instabilidade clínica. A integração entre protocolos bem definidos, consultorias por telemedicina e qualificação profissional contribui significativamente para um diagnóstico mais ágil, seguro e equitativo, especialmente em contextos de vulnerabilidade estrutural.

## Data Availability

Todo o conjunto de dados que dá suporte aos resultados deste estudo foi publicado no próprio artigo.

# Translation: Factores asociados al diagnóstico de muerte cerebral en hospitales del estado de Sergipe

**DOI:** 10.1590/1980-220X-REEUSP-2025-0014es

**Authors:** José Lucas dos Santos, Gabriella Santos Cisneiros, Jussiely Cunha Oliveira, Eduesley Santana Santos, Fernanda Gomes de Magalhães Soares Pinheiro

**Affiliations:** 1 Universidade Federal de Sergipe, Programa de Pós-Graduação em Enfermagem, Aracaju, SE, Brasil.; 2 Universidade Federal de Sergipe, Lagarto, SE, Brasil.; 3 Universidade Federal de Sergipe, Departamento de Enfermagem, Lagarto, SE, Brasil.; 4 Universidade Federal de Sergipe, Departamento de Enfermagem, Aracaju, SE, Brasil.

**Keywords:** Adulto, Muerte Encefálica, Epidemiología, Diagnóstico

## Abstract

**Objetivo:**

Analizar los factores asociados al diagnóstico de muerte encefálica (ME) en hospitales del Estado de Sergipe.

**Método:**

Estudio transversal realizado entre 2023 y 2024 en cuatro hospitales de Sergipe. Los participantes eran mayores de 18 años, de ambos sexos, con tres puntos en la Escala de Coma de Glasgow, lesión neurológica confirmada por tomografía craneal y ausencia de al menos dos reflejos del tronco.

**Resultados:**

El estudio incluyó 69 participantes y se observó una mayor prevalencia de individuos del interior (65%), con una mediana de edad de 52 años (42,0–68,0), mujeres (59%) y de etnicidad mixta (62%). Las principales causas neurológicas fueron el accidente cerebrovascular y el traumatismo craneoencefálico. Las personas de mayor edad y las personas con cáncer tienen menos probabilidades de que se les confirme la ME. La variable lugar de residencia se asoció significativamente con el diagnóstico.

**Conclusión:**

Se recomienda la integración de protocolos institucionales bien definidos, consultas de telesalud y capacitación profesional como estrategias que pueden conducir a un diagnóstico más rápido, seguro y equitativo en contextos de vulnerabilidad estructural.

## INTRODUCCIÓN

El concepto de muerte biológica se reformuló en 1981 sobre la base de la Ley de Determinación Uniforme de la Muerte (UDDA). Esta legislación definió la muerte como un evento caracterizado por el cese irreversible de las funciones circulatorias y respiratorias (criterios cardiopulmonares) o por el cese irreversible de todas las funciones cerebrales, incluidas las del tronco encefálico^(1)^.

A partir de la UDDA, Brasil y otros países adoptaron el concepto de muerte encefálica (ME). Así, la muerte encefálica se caracteriza por el cese irreversible de las funciones de los hemisferios cerebrales y del tronco encefálico, como consecuencia del compromiso de la circulación cerebral, secundario a una lesión conocida e irreversible^(2)^.

El mecanismo de la ME parte de una descompensación en la presión de perfusión cerebral (PPC), que es el resultado de la diferencia entre la presión arterial media (PAM) y la presión intracraneana (PIC). El aumento de la PIC reduce la PPC, lo que, en consecuencia, compromete la circulación en todo el cerebro. Estas disfunciones presóricas provocan daños cerebrales y favorecen la aparición de un edema progresivo, debido a una oxigenación inadecuada. Este proceso agrava aún más la PIC, pudiendo culminar en herniación, interrupción del flujo sanguíneo cerebral y necrosis. En las lesiones anóxicas, la hipoxia estimula la liberación de sustancias citotóxicas y el desarrollo de edema cerebral^(3)^.

Las principales causas de ME están relacionadas con eventos que afectan al cerebro, induciendo edema progresivo y posterior lesión irreversible. Entre las etiologías más comunes se destacan las enfermedades cerebrovasculares, el traumatismo craneoencefálico (TCE), la anoxia post-parada cardiorrespiratoria (PCR), las infecciones y los tumores del sistema nervioso central^(4)^.

En la mayoría de los países, el diagnóstico de ME se basa en la evaluación neurológica de las funciones de todo el cerebro y del tronco encefálico, asociada a la ausencia de movimientos respiratorios. En Estados Unidos, el diagnóstico de ME se rige por directrices y protocolos establecidos por organizaciones médicas de renombre^(5)^.

En Brasil, la Resolución del Consejo Federal de Medicina (CFM) n.º 2.173/2017 se formuló con el fin de definir los criterios para el diagnóstico de la muerte neurológica, dotando de rigor al proceso. La confirmación de esta lesión cerebral en el país se basa en la realización de dos exámenes clínicos con un intervalo mínimo de una hora para pacientes mayores de dos años, en la realización de una prueba de apnea y un examen complementario^(6)^; sin embargo, existen divergencias en su realización en todo el mundo. Ante esto, especialistas de todo el mundo acordaron la formulación de declaraciones que orientan los criterios neurológicos de la ME, con el fin de uniformarlos al máximo y garantizar una mayor rigurosidad en el proceso^(7)^.

La ausencia de protocolos institucionales para el diagnóstico de la ME, la falta de camas en UTI, la falta de recursos tecnológicos, la escasez de profesionales con experiencia y los conocimientos técnicos profesionales incipientes están estrechamente relacionados con las dificultades en el diagnóstico de la ME. Estos factores reflejan disparidades en la comprensión y el diagnóstico de esta afección, lo que contribuye a las deficiencias en la educación médica, la disponibilidad de recursos humanos y el uso de tecnologías adecuadas^(8)^.

En este contexto, el enfermero desempeña un papel esencial en la prevención de retrasos y en la viabilidad del diagnóstico de ME. La actuación continua de este profesional en la UTI permite la identificación precoz del empeoramiento neurológico, la comunicación eficaz con el equipo interdisciplinario y la preparación adecuada para los exámenes necesarios para el diagnóstico^(9)^.

Aunque el enfermero es reconocido en la gestión burocrática de la asistencia en ME, su eficacia en la asistencia y la gestión del equipo depende del desarrollo de habilidades técnicas y no técnicas, perfeccionadas a través de la experiencia y la educación continua^(10)^. La ausencia de esta mejora compromete su autoconfianza y la calidad de la asistencia, lo que puede provocar retrasos o fallos en el diagnóstico de la muerte cerebral^(11)^.

De este modo, la actuación proactiva del enfermero, a través de la formación continua del equipo, mejora los flujos institucionales. Este profesional también desempeña un papel estratégico en la orientación de los familiares, aclarando la irreversibilidad de la situación y reduciendo las resistencias que puedan comprometer el diagnóstico y la donación de órganos. La participación del enfermero en la elaboración de protocolos institucionales estandariza las conductas, agilizando y haciendo más asertivo el flujo en la confirmación de la ME^(11)^.

Este estudio llena un vacío importante en la literatura y ofrece una base para futuros estudios en el área, poniendo de manifiesto las dificultades estructurales y educativas que pueden influir en la conclusión de los protocolos. Para este estudio se planteó como cuestión principal «¿Qué factores clínicos, sociodemográficos y estructurales están asociados al diagnóstico de ME en los hospitales del estado de Sergipe?».

Así, el estudio se basa en la hipótesis de que residir en el interior puede estar asociado a la no confirmación del diagnóstico de ME, debido a múltiples factores que dificultan la conclusión de los protocolos. Entre ellos, destacan las limitaciones en la estructura tecnológica, la falta de disponibilidad de exámenes de imagen complementarios, la heterogeneidad institucional en términos de complejidad asistencial, la escasez de recursos humanos especializados y las deficiencias en la capacitación profesional. Estos factores, en conjunto, pueden contribuir a aumentar el tiempo necesario para confirmar el diagnóstico.

El objetivo de este estudio fue analizar los factores asociados al diagnóstico de ME en hospitales del estado de Sergipe.

## MÉTODO

### Diseño del Estudio

Se trata de un estudio transversal, guiado por las recomendaciones del *Strengthening the Reporting of Observational Studies in Epidemiology* (STROBE).

### Lugar

Este estudio se llevó a cabo en cuatro hospitales de Sergipe. En la capital, Aracaju, se realizaron dos, uno en un hospital de urgencias (H1) y otro en un hospital filantrópico (H2). En el interior, se realizó en un hospital universitario (H3), situado en la ciudad de Lagarto, y en un hospital general (H4), situado en la ciudad de Itabaiana. La selección de estas unidades hospitalarias se basó en la Resolución del CFM 2.173/2017, que exige la realización de un examen de neuroimagen para identificar la causa neurológica en pacientes elegibles para el protocolo de ME^(10)^. Por lo tanto, todos los hospitales participantes cuentan con servicio de neuroimagen las 24 horas y servicio de urgencias y emergencias.

### Población y Criterios de Selección

Se incluyeron participantes mayores de 18 años, de ambos sexos, que presentaban una puntuación de tres puntos en el ECG, con lesión neurológica confirmada mediante tomografía craneal realizada antes de la determinación de la muerte cerebral, y que presentaban la ausencia de dos de los siguientes reflejos: tos, pupilar y respiratorio.

Se excluyeron los participantes a los que se les descartó el diagnóstico de ME, que estaban bajo sedación y los que fallecieron antes de la realización de la tomografía craneal.

### Cálculo del Tamaño de la Muestra

Se utilizó como periodo de tiempo el año 2022 y los 212 protocolos iniciados en el estado y notificados a la Organización de Búsqueda de Órganos y Tejidos para Trasplantes^(12)^. Suponiendo una población finita, con un nivel de significación del 5%, un margen de error del 10% y una prevalencia del 50%, el tamaño de la muestra calculado fue de al menos 67 participantes, según la siguiente fórmula:

$$n=\frac{NZ_{\frac{\alpha }{2}}^{2}p(1-p)}{e^{2}(N-1)+Z_{\frac{\alpha }{2}}^{2}p(1-p)}$$

Donde *n* es el tamaño de la muestra, *N* es el tamaño de la población, *p* es la proporción esperada, y es el margen de error y $Z_{\frac{\alpha }{2}}^{2}$ es el cuadrado de la puntuación de la distribución normal asociada al nivel de significación *α*.

$$n=\frac{212\times {\left(1,965\right)}^{2}\times 0,5\times (1-0,5)}{{\left(0,10\right)}^{2}(212-1)+{\left(1,965\right)}^{2}\times 0,5\times (1-0,5))}\approx 67$$

### Recolección de Datos

El periodo de recolección de datos fue de agosto de 2023 a agosto de 2024 y se llevó a cabo mediante entrevistas con los familiares y tutores legales de los participantes, análisis de registros y historiales médicos de los pacientes. El instrumento de recolección fue estructurado y elaborado por el autor principal, basándose en formularios preexistentes de la Organización de Búsqueda de Órganos, y constaba de preguntas abiertas y cerradas.

El equipo responsable de la recolección de datos estaba compuesto por estudiantes de licenciatura en enfermería, posgrado y profesionales voluntarios. Todos los miembros recibieron formación teórica sobre el tema, los objetivos del estudio, los criterios de elegibilidad de los participantes y cómo rellenar el instrumento de recopilación de datos. Para calibrar a los investigadores, se realizaron recolecciones de prueba en los cuatro hospitales, con el objetivo de evaluar la estandarización de las recolecciones y la eficacia del instrumento en la obtención de los datos necesarios para el estudio.

Las entrevistas permitieron obtener información sobre variables sociodemográficas, como raza, estado civil, lugar de nacimiento, nivel educativo, número de hijos, profesión e ingresos, además del historial de enfermedades previas, cuando estos datos no estaban registrados en los expedientes de los participantes. Las etapas de la recolección de datos se detallan en la Figura 1.

**Figura 1:** Sistemática de la recolección de datos.

Los participantes fueron seleccionados mediante una búsqueda activa en las unidades de urgencias y las unidades de terapia intensiva de las instituciones. Tras verificar los criterios de inclusión, se aplicó el formulario de consentimiento libre y esclarecido al familiar o responsable legal mediante una entrevista.

### Análisis y Tratamiento de los Datos

El análisis estadístico se realizó utilizando medidas de tendencia central (media y mediana), dispersión (desvío estándar e intervalo intercuartílico) y distribución (frecuencia absoluta y porcentual).

La asociación entre variables categóricas se evaluó mediante la prueba de Chi-cuadrado, aplicando la prueba exacta de Fisher cuando fue apropiado. Para comparar las medianas entre dos o más muestras independientes, se utilizaron las pruebas de *Wilcoxon-Mann-Whitney* y *Kruskal-Wallis*, y para identificar específicamente qué grupos diferían entre sí se utilizó la prueba de *Dunn*, aplicada como *post hoc*.

Se empleó la regresión logística para modelar la relación entre variables predictivas y resultados binarios, utilizando modelos brutos (solo con la variable de interés), ajustados y multivariados, considerando solo las variables significativas en el modelo final. Se adoptaron como criterios de selección una significación inferior a 0,2, la ausencia de fenómenos de separación y la ausencia de multicolinealidad. El método de selección de variables utilizado fue el *backward*.

Los análisis estadísticos se realizaron en el entorno de programación R (versión 4.4.0) (R CORE TEAM, 2023), adoptando un nivel de significación del 5%.

### Aspectos Éticos

Este estudio fue aprobado por el Comité de Ética en Investigación (CEP) de la Universidad Federal de Sergipe el 21 de junio de 2023 con el número CAAE: 68731823.3.0000.5546 y número de dictamen 6.132.287.

## RESULTADOS

La muestra se definió a partir del seguimiento de pacientes que cumplían los criterios de inclusión (*n* = 231). De estos, se excluyeron los pacientes que fallecieron antes de la entrevista (*n* = 127), aquellos cuyo diagnóstico de ME fue descartado (*n* = 24) y los pacientes cuyos tutores no dieron su consentimiento mediante la firma del TCLE (*n* = 11), lo que dio un total de 69 participantes (Figura 2).

**Figura 2:** Flujograma de composición de la muestra y distribución de los grupos de resultados.

El estudio incluyó a 69 pacientes, con una mediana de edad de 52,0 [42,5; 67,8] años, con mayor prevalencia del sexo femenino (59%) y raza morena (62%). Los resultados mostraron además que el 65% de la muestra estaba compuesta por pacientes que residían en el interior del estado y la mitad tenía la educación primaria como nivel educativo más alto. En cuanto a la ocupación, el 34% eran jubilados y la mayoría de los participantes (56%) vivía con el salario mínimo (Tabla 1).

**Tabla 1:** Características sociodemográficas y clínicas de los casos confirmados y sospechosos de muerte cerebral Aracaju, SE, Brasil, 2024.

Características	N = 69
**Edad**	
Media (Desvío Estándar)	53,49 (17,29)
Mediana [25%;75%]	52,0 [42,0;68,0]
**Sexo, n/N (%)**	
Femenino	41/69 (59%)
Masculino	28/69 (41%)
**Raza, n/N (%)**	
Branca	16/64 (25%)
Negra	9/64 (14%)
Morena	39/64 (61%)
**Estado civil, n/N (%)**	
Casado	19/56 (34%)
Soltero	23/56 (41%)
Unión estable	11/56 (20%)
Viudo	3/56 (5%)
**Escolaridad, n/N (%)**	
Primaria	29/59 (49%)
Secundaria	21/59 (36%)
Superior	3/59 (5%)
Nunca estudió	6/59 (10%)
**Ocupación, n/N (%)**	
Jubilado	23/69 (33%)
Autónomo	17/69 (25%)
Funcionario privado	11/69 (16%)
Funcionario público	5/69 (7%)
Otros	13/69 (19%)
**Ingresos, n/N (%)**	
Dos a tres salarios	12/69 (17%)
No declaró	19/69 (28%)
Un salario	38/69 (55%)
**Institución de recolección, n/N (%)**	
H2	3/69 (4%)
H4	1/69 (1%)
H3	6/69 (9%)
H1	59/69 (86%)
**Unidad de internación, n/N (%)**	
Amarilla	3/69 (4%)
UTI	47/69 (68%)
Roja	19/69 (28%)
**Estilo de vida, n/N (%)**	
Etilismo	13/61 (21%)
Sedentarismo	31/61 (51%)
Tabaquismo	12/61 (20%)
Niega	3/61 (5%)
Otros	2/61 (3%)
**HAS, n/N (%)**	
No	14/47 (30%)
Si	33/47 (70%)
**DM, n/N (%)**	
No	32/47 (68%)
Si	15/47 (32%)
**Cáncer, n/N (%)**	
No	44/47 (94%)
Si	3/47 (6%)
**Cardiovascular, n/N (%)**	
No	41/47 (87%)
Si	6/47 (13%)

Siglas: n – Frecuencia absoluta; N – Datos válidos;% – Porcentaje; HTA – Hipertensión arterial sistémica; DM – Diabetes mellitus; UTI – UTI; H1 – Hospital de urgencias; H2 – Hospital filantrópico; H3 – Hospital universitario; H4 – Hospital general.

Los datos sobre las condiciones clínicas mostraron que la hipertensión arterial sistémica (HAS) era la comorbilidad más prevalente (70%). La diabetes mellitus (DM) se observó en el 32% y el sedentarismo era una condición presente en el 51% de la muestra. El H1 concentró el 86% de los pacientes, siendo la UTI la unidad de hospitalización más frecuente, con el 69% de los individuos hospitalizados.

En cuanto a la causa de la lesión cerebral (Figura 3), el accidente vascular cerebral hemorrágico (AVCH), el accidente vascular cerebral isquémico (AVCI) y el TCE fueron responsables de 18, 16 y 15 casos, respectivamente.

**Figura 3:** Causa de la lesión cerebral en casos de sospecha o confirmación de muerte encefálica.

En cuanto a los factores asociados al diagnóstico de ME (Tabla 2), la edad presentó una razón de prevalencia (RP) de 0,96 (IC 95%: 0,92–1,00; *p* = 0,044).

**Tabla 2:** Factores asociados al diagnóstico de ME basados en modelos brutos, ajustados y multivariados – Aracaju, SE, Brasil, 2024.

Características	Bruto		Ajustado		Multivariado	
	RP^1^ (IC 95%)^2^	Valor p	RP (IC 95%)	Valor p	RP (IC 95%)	Valor p
**Edad**	0,97 (0,94–1,00)	0,055	0,96 (0,92–1,00)	0,066	0,96 (0,92–1,00)	0,044
**Local de Residencia**						
Otras ciudades	–		–		–	
Grande Aracaju	5,38 (1,48–19,6)	0,011	5,49 (1,32–32,0)	0,031	5,26 (1,37–27,2)	0,025
**Cáncer**						
No	–		–		–	
Si	0,49 (0,17–1,45)	0,199	0,18 (0,04–0,73)	0,022	0,20 (0,04–0,78)	0,027

Fuente: Autores (2024).

Siglas: ^1^RP = Razón de prevalencia. ^2^IC = Intervalo con 95% de Confianza.

Por su parte, la variable cáncer presentó una fuerte asociación con la no confirmación del diagnóstico de ME (RP 0,18; IC 95%: 0,04–0,73; *p* = 0,022) y multivariante RP 0,20 (IC 95%: 0,04–0,78; *p* = 0,027). Por otro lado, a partir del análisis de la variable lugar de residencia, se observó que en todos los modelos estaba significativamente asociada a la confirmación del diagnóstico. Para el modelo ajustado se obtuvo una RP de 5,49 (IC 95%: 1,32–32,0; *p* = 0,031).

## DISCUSIÓN

Este estudio identificó una mayoría de individuos con una mediana de 52 años, predominio del sexo femenino, raza morena, estado civil soltero y nivel educativo hasta la educación primaria (Tabla 1). Estos resultados concuerdan con los datos encontrados en una investigación realizada con 60 donantes potenciales de órganos en Natal/RN^(13)^.

Sin embargo, otros resultados destacan una mayor prevalencia en individuos de sexo masculino, como por ejemplo, el estudio estadounidense que encontró un predominio masculino (56,47%) entre los casos de ME^(2)^. Además, otro estudio mostró una mayor prevalencia en hombres (61,99%) en comparación con las mujeres (38,01%)^(14)^.

Los hallazgos de las principales causas de ME en este estudio refuerzan otros resultados en el panorama mundial. En Turquía, entre 2020 y 2023, se verificó que las principales causas de ME fueron el AVC y la hemorragia intracraneal, que representaron el 45,6% y el 47% de los casos. El TCE, la segunda causa, fue responsable del 20,7% y el 21% de los casos en los años estudiados, mientras que el AVC fue la tercera, con una prevalencia del 17,8% y el 18%, respectivamente^(14)^.

En Brasil, el panorama es similar al de otros países. El AVC y el TCE se encuentran entre las principales causas, sumando más del 90% de los casos^(15)^. Otras evidencias revelaron hemorragia subaracnoidea en el 58,2% de los casos, mientras que el AVC representó el 7,1% y el TCE el 19,4% de los diagnósticos^(16)^.

Al comparar los resultados de este estudio con los encontrados en el último estudio realizado en Sergipe, se observa un cambio en la prevalencia de las causas neurológicas de la ME. En el estado, el TCE ya era la principal causa de la ME, representando el 68,3% de los casos, seguido por el AVC con el 28,6%, mientras que los tumores primarios del sistema nervioso central representaban el 3,1% de la muestra^(17)^.

En Brasil, a pesar de que el TCE se encuentra entre las principales causas, en el año 2023, según datos de la Asociación Brasileña de Trasplantes de Órganos (ABTO), la ME pasó a ser más prevalente en pacientes con AVC (52% de las causas), seguido por el TCE (31% de las causas) y la anoxia posparada cardiorrespiratoria^(18)^. La transición observada indica que las causas pueden estar condicionadas por el perfil epidemiológico, los hábitos de vida y el envejecimiento de la población, este último asociado a enfermedades cerebrovasculares, ya que son más prevalentes en pacientes de edad avanzada^(19)^.

Los factores asociados al diagnóstico de ME (Tabla 2) indican que los pacientes de mayor edad tenían un 4% menos de probabilidades de obtener un diagnóstico confirmado. En contraste con estos hallazgos, un estudio similar encontró una edad media de 48 años entre los pacientes con ME confirmada, y al menos el 10% de los casos afectaban a personas menores de 18 años^(2)^.

Esta asociación entre la edad avanzada y una menor probabilidad de confirmación puede deberse a la alta prevalencia de TCE en individuos más jóvenes, ya que este traumatismo constituye la segunda etiología principal de la ME^(18)^. La mayor frecuencia de esta causa en pacientes jóvenes repercute directamente en la edad media de la población afectada, reforzando el perfil de edad predominante entre los casos confirmados.

Además, los pacientes de mayor edad tienden a tolerar menos dos eventos fisiopatológicos que se observan con frecuencia en la ME: la inestabilidad hemodinámica y la hipertensión intracraneal (HIC). Los cambios fisiológicos relacionados con el envejecimiento, como la sarcopenia, una afección prevalente con la edad^(20)^, agravan el pronóstico de las víctimas de TCE. La pérdida de masa muscular contribuye al estado hipercatabólico sistémico y cerebral resultante de la respuesta al estrés, lo que intensifica la lesión neurológica y empeora el desenlace clínico^(21)^.

El edema cerebral, principal consecuencia de la HIC, tiende a agravarse ante otros factores clínicos y comorbilidades comunes en este grupo de edad, como la hipernatremia y las alteraciones glicémicas derivadas de la DM. Estos factores aumentan el riesgo de edema cerebral y de lesión axonal difusa^(22)^. Ante esta situación, la asociación de comorbilidades y alteraciones fisiopatológicas compromete la estabilidad clínica de los pacientes de edad avanzada, reduciendo su capacidad para soportar las pruebas confirmatorias de ME que evolucionan hacia la PCR antes de la finalización del protocolo diagnóstico^(23)^.

La identificación de neoplasias durante la investigación de ME ha demostrado reducir hasta en un 92% las posibilidades de confirmación diagnóstica. Aunque poco explorada en la literatura, la relación entre el cáncer y la ME se ha considerado en estudios en los que los pacientes oncológicos sometidos a cuidados paliativos pueden recibir tratamientos menos intervencionistas, lo que puede retrasar o incluso imposibilitar la conclusión del protocolo diagnóstico. Cabe destacar que los pacientes oncológicos y aquellos en ME suelen seguir directrices clínicas distintas, lo que puede afectar directamente al diagnóstico de la lesión neurológica^(24)^.

La coexistencia de estos dos diagnósticos constituye una contraindicación absoluta para la donación de órganos. En la práctica clínica, esta limitación reduce el compromiso de los equipos médicos en la realización de todas las etapas del protocolo de ME, a pesar de que es un derecho del paciente y de sus familiares obtener un diagnóstico definitivo^(6)^.

De manera complementaria, son frecuentes los relatos de sobrecarga emocional y intenso sufrimiento psicológico, tanto por parte de los familiares como de los propios pacientes. Ante la gravedad del cuadro y la posibilidad de que el paciente no tolere todas las etapas de los exámenes clínicos y de imagen, en muchas situaciones el equipo opta por preservarlos de este proceso, priorizando las medidas de confort y eligiendo la limitación terapéutica como conducta principal con los familiares^(25)^.

La región de residencia reveló una asociación significativa con la confirmación de ME. Específicamente, los residentes de la Gran Aracaju presentaron un aumento del 449% en las posibilidades de confirmación de ME, en comparación con los que residen en otras localidades. Este hallazgo indica disparidades en el acceso y la eficacia del diagnóstico entre las regiones.

Este resultado plantea interrogantes sobre la eficacia de uno de los principios básicos de la Red de Atención a la Salud, la integralidad de la asistencia sanitaria. La desproporción observada puede estar relacionada con fallos en la articulación entre los servicios del interior y de la capital, lo que dificulta la realización oportuna de exámenes complementarios y la aplicación adecuada de los protocolos de ME. La fragmentación de la red y la concentración de recursos en los centros urbanos repercuten negativamente en la equidad en el acceso a los servicios especializados, lo que puede remitir a las dificultades encontradas fuera de la Gran Aracaju^(26)^.

Por otra parte, la literatura señala la asociación entre el lugar de residencia del paciente y la prevalencia de casos de ME. En Campo Grande/MS, por ejemplo, se observó una mayor prevalencia en la capital, con 39 casos (69,7%), en comparación con 17 casos (30,3%) en el interior^(27)^. Esta diferencia puede justificarse por la concentración de mejores centros de salud en las capitales, con mejor infraestructura, más tecnologías, equipos sofisticados y profesionales especializados, factores que aumentan las posibilidades de éxito en la realización de los diagnósticos^(28)^.

En Sergipe, el único centro público de referencia para el tratamiento de los casos de ME es el hospital público de urgencias de la capital, que cuenta con camas de reserva y un equipo que trabaja las 24 horas del día en la búsqueda activa de casos, la aplicación de los protocolos y la viabilidad de las políticas de donación de órganos. Esta estructura promueve una mayor agilidad en el diagnóstico y la gestión de los casos, lo que puede haber influido en los resultados de esta asociación.

La pérdida de posibles participantes antes de la firma del TCLE debido a la muerte por inestabilidad hemodinámica, así como la falta de conocimiento de los familiares sobre ME, dificultaron la inclusión de participantes en este estudio. Sin embargo, el estudio llena importantes lagunas en la literatura, proporcionando una visión global de los factores asociados al diagnóstico de ME en Sergipe. A partir de los resultados, es posible proponer mejoras en los procesos de diagnóstico en el estado, así como optimizar las políticas públicas de salud orientadas a la donación de órganos.

En este contexto, es esencial que el enfermero desempeñe el papel de líder y educador, en colaboración con el equipo multidisciplinario, con el fin de transformar esta realidad, junto con la sensibilización de la población, especialmente de los familiares de los pacientes, sobre el significado de la ME y su importancia para las políticas públicas de donación de órganos.

La educación continua de los profesionales de enfermería ha demostrado ser una forma eficaz de mejorar la calidad de la atención prestada y, en el contexto de la ME, esta estrategia mejora la comprensión del proceso diagnóstico, la visión clínica y la detección precoz de posibles casos, además de contribuir a la eficacia de las políticas de donación de órganos^(29)^. Reduce el frecuente retraso en la apertura de los protocolos por desconocimiento del manejo adecuado del paciente con sospecha de ME, ya sea por descontrol de la temperatura, inestabilidad hemodinámica o parámetros gasométricos que impiden la apertura del protocolo, lo que se traduce en una mayor agilidad y rapidez en el diagnóstico^(30)^.

## CONCLUSIÓN

La confirmación del diagnóstico de ME está directamente influenciada por la proximidad de los pacientes a centros de salud bien estructurados, lo que favorece la agilidad y la precisión del proceso. Sin embargo, en regiones remotas y con acceso limitado a especialistas, es necesario adoptar estrategias innovadoras para garantizar la eficiencia diagnóstica, minimizando las desigualdades en la atención.

Entre estas estrategias, destaca la creación de protocolos institucionales con listas de verificación y flujos basados en la evidencia, con el objetivo de promover la estandarización, la precisión y la agilidad en el diagnóstico. Estas medidas pueden reducir los errores y optimizar el tiempo de confirmación de la ME, incluso en unidades con recursos limitados. De manera complementaria, el uso de la telesalud como herramienta innovadora puede mitigar los impactos de la escasez de especialistas al permitir consultas remotas con neurólogos e intensivistas, lo que posibilita la validación de los criterios clínicos, la realización del diagnóstico y el análisis de exámenes, como la tomografía.

Otro aspecto esencial es la inversión continua en la capacitación del personal de enfermería, orientada a la identificación temprana de los casos y al monitoreo de los signos de inestabilidad clínica. La integración entre protocolos bien definidos, consultas por telemedicina y la cualificación profesional contribuye significativamente a un diagnóstico más ágil, seguro y equitativo, especialmente en contextos de vulnerabilidad estructural.

## Data Availability

Todo el conjunto de datos que respalda los resultados de este estudio fue publicado en el propio artículo.
